# Classification of the Phases of Diabetes

**Published:** 1913-07-12

**Authors:** 


### DIABETES AND GLYCOSURIA.

### Classification of the Phases of Diabetes.

With the accumulation of experience concerning the condition which is characterised by the appearance of sugar (glucose) in the urine, physicians long ago began to recognise that in some cases very simple dietary, and other, treatment sufficed to restore the patient to health, whereas in others all the resources of the Pharmacopœia proved quite unable to do so. Accordingly, a habit sprang up a few years ago of dubbing these milder curable cases "alimentary glycosuria," while reserving the term "diabetes" for the more serious and intractable forms. This differentiation probably received more favour than it otherwise might have done, because the ominous import of the word diabetes is fairly common knowledge with the public; and any euphemistic synonym was not unnaturally made welcome. In Dr. Garrod's Lettsonian Lectures last year he brought forward a mass of evidence in support of the thesis that no such double nomenclature is admissible; that the condition of voiding glucose in the urine is not a single definite circumscribed disease; and that the disturbance of metabolism which leads to hyperglycæmia, and so to glycosuria, has its origin in a variety of causes.*

Pathologically classified, these causes can be divided into those which produce an excess of sugar in the blood; this is then filtered off by the kidneys and appears in the urine. (It has to be remembered that minute quantities of sugar are present in normal urine, but not enough to answer the usual reduction test.) In a second class stand those cases in which the sugar contained in the blood is normal, or below normal; in these the kidneys must be to blame for failing to retain the normal percentage which in health they can succeed in doing.

But pathological classification does not at present carry us sufficiently far to be helpful in the treatment and prognosis of diabetes. From a clinical standpoint cases of metabolic glycosuria can be sifted into three main varieties: those in which glycosuria does not occur in normal circumstances, but may be induced by an indulgence in sugar or sugar-containing foods, which would not suffice to disturb the urinary content of an ordinary individual; those in which spontaneous glycosuria occurs intermittently, apart from dietary peculiarities; and those in which glycosuria is firmly established, and cannot be got rid of, or only very temporarily, by the most rigid anti-diabetic regimen. The first of these is the condition to which the label of alimentary glycosuria has been frequently fastened. But Dr. Garrod draws attention to the fact that there is no hard-and-fast line between these classes, and that individual patients may pass from one class to another. In other words, there is no true distinction between diabetes and glycosuria, although those terms do certainly cover a collection of diseases of diverse origins having the presence of sugar in the urine as their leading feature.

Dr. Garrod states that a normal person can consume somewhere about five to seven ounces of grape-sugar without thereafter passing it in the urine. Not many adult people, it may be supposed, consume anywhere approaching such a surfeit; so that their tolerance is never strained to the region of its limits. But children are often given to gorging themselves on sugary foods, and may therefore, if allowed to exercise their own lack of discretion, do themselves an injury by imprudent sweetmeat- or sugar-eating. The writer knows of a case in which an adult was discovered to be passing sugar in the urine, and admitted a passion for children's sweets, which had long been gratified to great excess. When the habit was discontinued, the urine returned to a normal condition after no very great interval of time.

Starchy foods are, as is well known, very provocative of glycosuria in those with a tendency to diabetes. This is due to the fact that they are converted into soluble sugar during the processes of digestion. In healthy people it seems impossible to set up glycosuria by any dose of starch which can be taken, as the conversion into sugar does not take place sufficiently rapidly to tax the tolerance. When a meal of starch produces glycosuria, a definitely morbid condition of the metabolism exists. He who excretes small quantities of sugar after a meal rich in carbohydrates differs only in degree from him who has pronounced and advanced diabetes; and the interval between them is bridged by intermediate cases of all grades. It is a matter of dispute, says Dr. Boyd,† whether lowering of glucose tolerance is to be regarded as the initial manifestation of that error of carbohydrate metabolism which culminates in diabetes, or of a wholly different significance from glycosuria "ex amylo" and spontaneous glycosuria. But it may be said that when a patient shows lowered glucose tolerance in the course of an acute disease, it does not in the least follow that he is likely to develop diabetes later on.

Many of the "ductless glands" seem to have peculiar influences upon the metabolic processes which lead to glycosuria, possibly through the action of enzymes. Though not strictly speaking ductless, the pancreas has long been held to form an internal secretion quite distinct from the pancreatic juice, and to have a great deal to do with diabetes. Exophthalmic goitre, again, Dr. Garrod points out, is sometimes associated with diabetes, and more often still with a distinct reduction of glucose tolerance. Moreover, the two conditions of lowered glucose tolerance and actual glycosuria can be induced by the medicinal use of thyroid extract. Out of eleven cases of myxœdema under thyroid medication, glycosuria was actually found in four.

* An excellent summary of Dr. Garrod's Lectures can be found in the *Medical Annual*, 1913, p. 259. The originals are published in the *Lancet*, 1912, i., 483.

† *Medical Annual, loc. cit.*

In the same way the pituitary body has an influence on carbohydrate metabolism. Glycosuria may occur in acromegaly, and is said to be curiously capricious, appearing or disappearing without sufficient obvious reason. The suprarenals, again, have an influence, though as yet it is very obscure, upon the production of glycosuria, or possibly of hyperglycæmia primarily.

Diabetes, then, appears to be merely the maximal phase of a series, rising by gradual steps from the normal of metabolism; just as myxœdema is the culminating point of the almost insensible grades of hypothyroidism. With the possible exception of so-called renal glycosuria, there is no such thing as non-diabetic glycosuria, although there are many varieties which lack the sinister significance associated with the name of diabetes. The differences are rather of degree than of kind.

